# The reliability and feasibility of non-contrast adenosine stress cardiovascular magnetic resonance T1 mapping in patients on haemodialysis

**DOI:** 10.1186/s12968-020-00634-y

**Published:** 2020-06-08

**Authors:** Federica E Poli, Gaurav S Gulsin, Daniel S March, Ahmed MSEK Abdelaty, Kelly S Parke, Joanne V Wormleighton, Gerry P McCann, James O Burton, Matthew PM Graham-Brown

**Affiliations:** 1 Department of Cardiovascular Sciences, University of Leicester and the NIHR Leicester Cardiovascular Biomedical Research Unit, Glenfield Hospital, Leicester, LE1 9HN UK; 2 NIHR Leicester Biomedical Research Centre, University Hospitals of Leicester NHS Trust, Leicester, UK; 3 John Walls Renal Unit, University Hospitals Leicester NHS Trust, Leicester, UK; 4 National Centre for Sport and Exercise Medicine, School of Sport, Exercise and Health Sciences, Loughborough University, Loughborough, UK

**Keywords:** Coronary artery disease, Myocardial ischaemia, End-stage renal disease, Haemodialysis, Stress T1, Feasibility, Reproducibility, Reliability

## Abstract

**Background:**

Identifying coronary artery disease (CAD) in patients with end-stage renal disease (ESRD) is challenging. Adenosine stress native T1 mapping with cardiovascular magnetic resonance (CMR) may accurately detect obstructive CAD and microvascular dysfunction in the general population. This study assessed the feasibility and reliability of adenosine stress native T1 mapping in patients on haemodialysis.

**Methods:**

The feasibility of undertaking rest and adenosine stress native T1 mapping using the single-shot Modified Look-Locker inversion recovery (MOLLI) sequence was assessed in 58 patients on maintenance haemodialysis using 3 T CMR. Ten patients underwent repeat stress CMR within 2 weeks for assessment of test-retest reliability of native T1, stress T1 and delta T1 (ΔT1). Interrater and intrarater agreement were assessed in 10 patients. Exploratory analyses were undertaken to assess associations between clinical variables and native T1 values in 51 patients on haemodialysis.

**Results:**

Mean age of participants was 55 ± 15 years, 46 (79%) were male, and median dialysis vintage was 21 (8; 48) months. All patients completed the scan without complications. Mean native T1 rest, stress and ΔT1 were 1261 ± 57 ms, 1297 ± 50 ms and 2.9 ± 2.5%, respectively. Interrater and intrarater agreement of rest T1, stress T1 and ΔT1 were excellent, with intraclass correlation coefficients (ICC) > 0.9 for all. Test-retest reliability of rest and stress native T1 were excellent or good (CoV 1.2 and 1.5%; ICC, 0.79 and 0.69, respectively). Test-retest reliability of ΔT1 was moderate to poor (CoV 27.4%, ICC 0.55). On multivariate analysis, CAD, diabetes mellitus and resting native T1 time were independent determinants of ΔT1 (β = − 0.275, *p* = 0.019; β = − 0.297, *p* = 0.013; β = − 0.455; *p* < 0.001, respectively).

**Conclusions:**

Rest and adenosine stress native T1 mapping is feasible and well-tolerated amongst patients with ESRD on haemodialysis. Although rater agreement of the technique is excellent, test-retest reliability of ΔT1 is moderate to poor. Prospective studies should evaluate the relationship between this technique and established methods of CAD assessment and association with outcomes.

## Background

Patients with chronic kidney disease and end stage renal disease (ESRD) experience excess mortality from cardiovascular disease [1].. Developing safe and robust investigations that can reliably identify coronary artery disease (CAD) to enable optimal risk stratification, intervention and management is a priority for this population [2]. Contrast-enhanced cardiovascular magnetic resonance (CMR) is a validated and established technique for the assessment of CAD, allowing evaluation of both myocardial perfusion defects and infarct burden [3, 4]. However, this requires administration of gadolinium-based contrast, which is contraindicated in patients with advanced kidney disease, due to the rare but serious risk of nephrogenic systemic fibrosis [5].

Native T1 mapping is a non-contrast parametric mapping technique on CMR that allows quantitative tissue characterisation on a pixel-by-pixel basis [6]. T1 relaxation time increases with higher free water content [7]. The total myocardial T1 value is derived from the contribution of the intracellular, extracellular and blood compartments [8]. Under normal circumstances, adenosine vasodilator stress causes an increase in myocardial blood volume and, consequently, an increase in myocardial native T1 [9]. Myocardial stress T1 reactivity (ΔT1) is, therefore, thought to represent the percentage increase in myocardial blood volume during vasodilator stress [10]. As disturbances in absolute quantification of myocardial blood volume changes can detect significant coronary artery stenoses [11], a blunted ΔT1 could be a non-invasive non-contrast measure to differentiate ischaemic, infarcted and normal myocardium [10]. Adenosine stress T1 mapping has recently been demonstrated to accurately detect obstructive CAD and microvascular dysfunction in patients with stable angina and healthy subjects [12]. However, this technique has never been tested in a population with ESRD. These patients have a complex cardiovascular disease phenotype, marked by both coronary and non-coronary disease-related processes, high levels of interstitial myocardial fibrosis [13, 14] and significantly higher myocardial native T1 times compared to control subjects [15, 16]. Whether stress T1 mapping could be an appropriate test to assess CAD in this patient group is therefore not known.

In this study we assessed the feasibility of non-contrast rest and stress T1 mapping in patients with ESRD on haemodialysis. We aimed to assess the ΔT1, its reliability with test-retest measurements, interrater and intrarater agreement in patients on haemodialysis and we conducted an exploratory analysis to investigate associations between clinical variables and ΔT1.

## Methods

### Study population

Patients with ESRD on haemodialysis were recruited as part of the CYCLE-HD study (ISRCTN11299707). The study was approved by the NHS Research Ethics Committee East Midlands (Northampton; REC ref.: 14/EM/1190). All patients provided written informed consent to participate in the study. Adult patients undergoing in-centre maintenance haemodialysis for at least 3 months were eligible for inclusion in the study, provided they were able to undertake exercise and undergo CMR scanning. Inclusion and exclusion criteria are as previously described [17].

### CMR image protocol

CMR imaging was performed on a 3 T CMR platform (Skyra, Siemens Healthineers, Erlangen, Germany) with an 18-channel phased-array anterior coil. To ensure consistency of fluid status, all patients underwent CMR assessment on a non-dialysis day, not after the two-day break, between 18 and 24 h of the last haemodialysis session. The same standardised CMR protocol was used for all study participants as previously described [16], in line with internationally recognised standards [18]. Briefly, mid-ventricular, short-axis native T1 maps were acquired using the single-shot Modified Look-Locker inversion recovery (MOLLI) sequence [19] with the 3(3)3(3)5 sampling pattern. Images were acquired at end-expiration, using free-breathing with motion correction and electrocardiogram (ECG)-gating. Typical parameters were: slice thickness 8.0 mm, field of view 300 × 400 mm, flip angle 50^0^, minimum T1 120ms, inversion-time increment 80 ms. The MOLLI sequence was chosen due to local expertise [20] and the excellent reliability demonstrated in this patient population at 3 T [21]. All participants were asked to avoid caffeine on the day of their scan. At the study visit, they had the choice to undergo adenosine stress scanning or not. If they agreed to it, after the acquisition of rest images, adenosine was infused intravenously at an initial dose of 140 μg/kg/min for 3 minutes. Adequate haemodynamic response was defined as the occurrence of two of the following: ≥10% increase in heart rate; ≥10 mmHg decrease in systolic blood pressure; reporting of typical symptoms (e.g. flushing, shortness of breath, chest discomfort). In case of inadequate haemodynamic response, adenosine dose was increased incrementally to 170 μg/kg/min and to a maximum of 210 μg/kg/min [22]. Stress T1 maps were acquired in a mid-ventricular short-axis slice in the same slice position as resting T1 maps.

### CMR image analysis

**Fig. 1:**
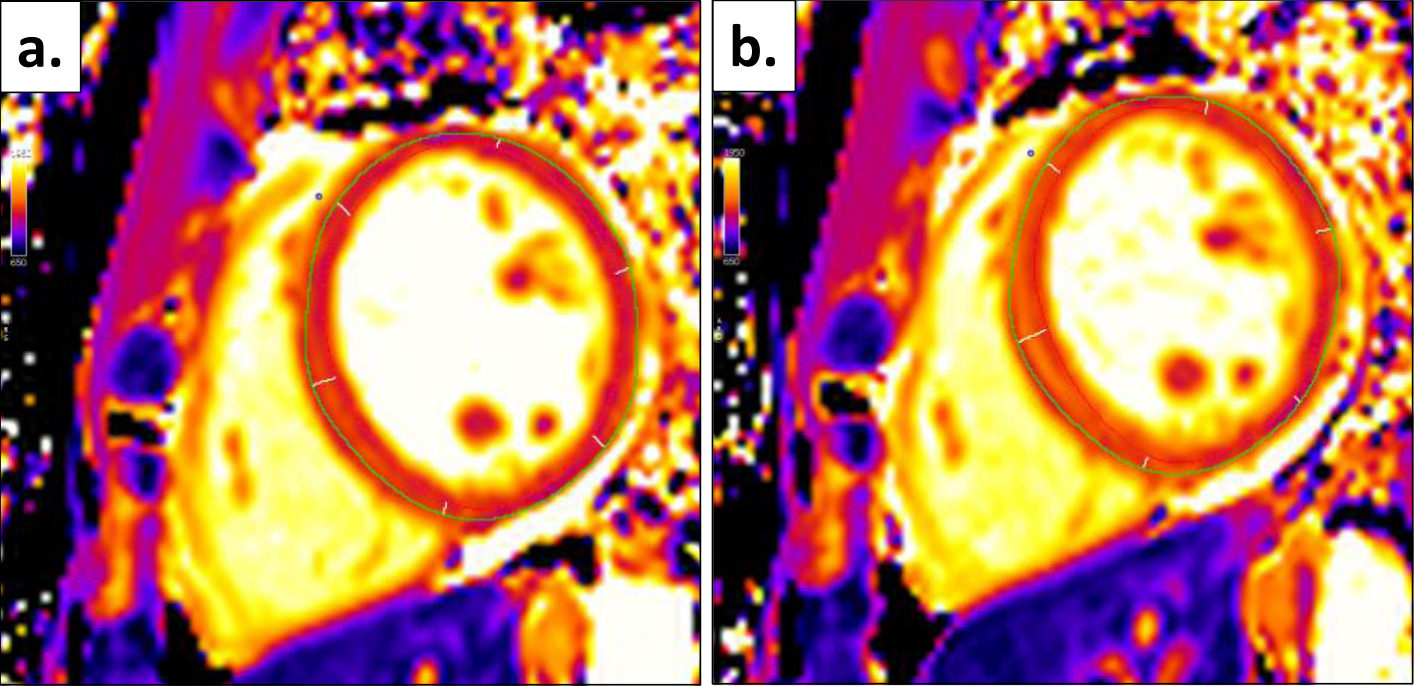
T1 map analysis and segmentation of mid-ventricular slice (**a**) at rest and (**b**) during adenosine stress, based on definition of right ventricular insertion point (blue dot)

All scans were anonymised and analysed offline by blinded observers. The software package CMR^42^ (version 5.10.1, Circle Cardiovascular Imaging, Calgary, Alberta, Canada) was used for both left ventricular (LV) and native T1 analyses. LV quantification was performed by a single reader (MGB) as previously described [16]. A separate reader completed T1 map analysis (FP) as previously described [21]. Briefly, endocardial and epicardial contours were manually drawn for native T1 maps. Care was taken to allow sufficient margins of separation between the myocardium and other tissue interfaces to avoid partial blood pool volume or epicardial fat contaminations. After the definition of the anterior right ventricular insertion point, the mid-ventricular short axis slice was automatically divided into 6 segments, according to the American Heart Association 16-segment model (Fig. 1). Infarcted segments, which were too thin to contour (with the inability to differentiate myocardium from blood pool) or with clear akinesis on cine imaging were excluded, as were myocardial segments with artefact. If certain segments required exclusion, the average myocardial T1 time was calculated as the mean of the remaining segments. If no segments required exclusion, the analysis software generated the average myocardial T1 value using a three-parameter least-squares fitting technique with heart rate correction. ΔT1 was calculated, as previously described using the following formula [8, 10, 12, 23–25]:

$$ \Delta  T1=\frac{stress\ T1- rest\ T1}{rest\ T1}\times 100 $$

Image quality was graded as excellent, moderate or non-analysable.

An additional, descriptive analysis comparing regional wall motion (RWM) scores to ΔT1 scores was completed. The mid-ventricular, short-axis LV cine image corresponding to the mid-ventricular native T1 maps for each subject were scored for RWM abnormalities. Each segment was scored 1 (normal), 2 (hypokinetic), 3 (akinetic) or 4 (dyskinetic) (AA). Segments were then compared to corresponding ΔT1 segment for analysis.

### Test-retest reliability, interrater and intrarater agreement

Ten patients underwent an identical CMR scan within 2 weeks of the baseline scan as part of a reliability sub-study. Test-retest reliability was assessed by a single, blinded observer (FP). Interrater agreement was assessed by analysis of the same 10 scans by two independent, blinded readers (FP; MGB). Intrarater agreement was assessed by re-analysis of 10 scans by a single blinded reader (FP), 1 month apart.

This sample size of 10 was supported by a power calculation previously done to detect a 2.5% difference in native T1 between test-retest scans with 90% power [21].

### Exploratory analyses

Exploratory analyses were undertaken to establish potential associations between ΔT1 and clinical indicators of CAD, including age, sex, heart rate (HR), blood pressure (BP), smoking status, previous history of CAD, diabetes mellitus, previous renal transplant, hypertension and dyslipidaemia. All CMR images used for the exploratory analysis were acquired as part of the baseline assessment, with patient demographic details, comorbidities, medications, haematological and biochemical data were recorded prospectively. Total time on renal replacement therapy (RRT), time with a functioning kidney transplant and haemodialysis vintage were prospectively collected and calculated from electronic hospital records. The presence of CAD was defined as any of: reported diagnosis of CAD on baseline assessment; fixed or reversible ischaemia on myocardial perfusion scan; significant stenosis on invasive coronary angiography; infarcted myocardium on CMR; previous coronary revascularisation (coronary artery bypass graft or percutaneous coronary intervention). Individuals who had a non-analysable stress scan were excluded from this analysis.

### Statistical analysis

Statistical analyses were performed using SPSS (v 25.0, Statistical Package for Social Sciences, International Business Machines, Inc.,, Armonk, New York, USA) and GraphPad Prism (v 8.1.1, GraphPad Software, Inc., La Jolla, California, USA). Categorical data are reported as frequency (%) of observation. Normality was assessed using histograms and Shapiro-Wilk tests for continuous data. Normally distributed data are expressed as mean ± standard deviation (SD). Non-normally distributed data are expressed as median (P25, 75). Test-retest reliability, interrater and intrarater agreement were assessed using coefficients of variability (CoV), intraclass correlation coefficients (ICC) and Bland-Altman analyses [26]. Reliability was considered excellent for CoV < 10%, ICC > 0.90; good for CoV < 15%, ICC 0.75–0.90; moderate for CoV < 20%, ICC 0.50–0.74 and poor for CoV > 20%, ICC < 0.50. Comparisons between groups were made using independent sample *t*-tests data and Mann-Whitney *U* tests for normally and non-normally distributed data, respectively. Differences between nominal variables were assessed using Chi-squared and Fisher’s exact tests. Correlations between variables were assessed with Pearson’s and Spearman’s rank analysis for normally and non-normally distributed data, respectively. Multivariable linear regression analyses were conducted to assess independent determinants of ΔT1. Variables inputted into regression models were limited to avoid overfitting. Statistical significance level was defined as a two-tailed *p*-value < 0.05. The exploratory analysis comparing RWM to regional ΔT1 are presented as descriptive statistics due to the imbalance in numbers between groups.

## Results

### Feasibility and analysability

**Fig. 2:**
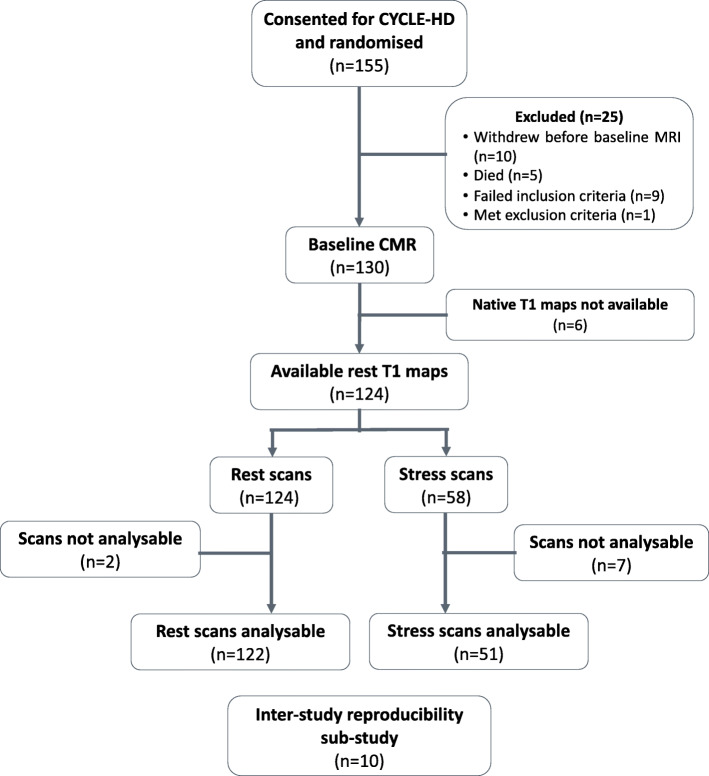
Consort diagram

The baseline scans of 58 of the 130 participants of the CYCLE-HD study agreed to have both rest and adenosine stress native T1 mapping. Baseline characteristics are shown in Table 1. Additional haematological, biochemical and medication data can be found in Appendix 1. A consort diagram showing patients included in the study is shown in Fig. 2. Only two of the 124 (1.6%) resting T1 maps were not analysable due to poor image quality. Image quality was either excellent (*n* = 93) or moderate (*n* = 29) for the remaining 122 resting T1 maps. Seven of 58 (12%) stress T1 maps were not analysable. Image quality for the remaining 51 stress T1 maps was either excellent (*n* = 32) or moderate (*n* = 18). All patients with analysable stress T1 maps also had analysable matched resting T1 maps. Of the 58 participants who underwent stress testing, six required an increase in adenosine dose. Only one participant had an inadequate haemodynamic response despite maximal dose of adenosine. All patients tolerated and successfully completed the scan with no complications necessitating termination of the scan. After the administration of adenosine, there was a significant increase in HR (71 ± 12 bpm versus 85 ± 14 bpm; *p* < 0.001) and a significant drop in systolic BP (152 ± 34 mmHg versus 136 ± 35 mmHg; *p* < 0.001). 86% of the patients experienced mild symptoms (Table 2). There was no significant association between heart rate and native T1 values at rest (*r* = − 0.096; *p* = 0.476) or during stress (*r* = − 0.118; *p* = 0.387).

**Table 1:** Baseline characteristics

	*n* = 58
Age (years)	55 ± 15
Male sex, n (%)	46 (79)
Ethnicity, n (%)	
White	24 (41.4)
Asian	28 (48.3)
Black, Mixed, Other	6 (10.3)
BMI (kg/m^2^)	26.4 (23.0; 29.7)
Systolic blood pressure (mmHg)	144.0 ± 39.5
Diastolic blood pressure (mmHg)	64.7 ± 16.8
Active on transplant list	18 (31)
Previous transplant, n (%)	18 (31)
Total RRT time (months)	32.0 (11.8; 95.0)
Haemodialysis vintage (months)	21.0 (8.0; 48.3)
Comorbidities	
Coronary artery disease	15 (25.9)
Previous myocardial infarction	9 (15.5)
Coronary revascularisation	7 (12.1)
Hypertension	37 (63.8)
Diabetes mellitus	24 (41.4)
Dyslipidaemia	13 (22.4)
Heart failure	0
Peripheral vascular disease	0
Atrial fibrillation	2 (3.4)
Cerebrovascular disease	2 (3.4)
Asthma/COPD	0
CMR parameters	
Global native T1 rest (ms)	1261 ± 57
Global native T1 stress (ms)	1297 ± 50
Myocardial ΔT1 (%)	2.9 ± 2.5
LV mass index (g/m^2^)	60.1 (52.8; 76.8)
LV end-diastolic volume index (ml/m^2^)	87.6 (67.6; 116.5)
LV end-systolic volume index (ml/m^2^)	37.6 (26.9; 51.6)
LV ejection fraction (%)	54.8 ± 8.3
LV mass / LV end-diastolic volume	0.73 ± 0.17

Categorical variables presented as number (%). Normally distributed data presented as mean ± SD. Non-normally distributed data presented as median (P25, 75)

*BMI* Body mass index, *LV* Left ventricular, *COPD* Chronic obstructive pulmonary disease, *LV* Left ventricular, *RRT* Renal replacement therapy

**Table 2:** Summary of stress response

	*n* = 58
Baseline HR (beats/min)	71 ± 12
Maximal HR during stress (beats/min) *[n = 57]*	85 ± 14
HR change (beats/min) *[n = 57]*	14 ± 8
Achieved change in HR > 10%, n (%) *[n = 57]*	49 (86)
Baseline systolic BP (mmHg) *[n = 41]*	152 ± 34
Minimal systolic BP during stress (mmHg) *[n = 40]*	136 ± 35
Systolic BP change (mmHg) *[n = 40]*	−15 ± 17
Achieved decrease in systolic BP > 10 mmHg, n (%) *[n = 40]*	28 (70)
Symptoms, n (%) *[n = 43]*	37 (86)
Required adenosine dose increase, n (%)	6 (10)

Categorical variables presented as number (%). Normally distributed data presented as mean ± SD. Non-normally distributed data presented as median (P25, 75). *HR* Heart rate, *BP* Blood pressure

### Reliability

**Fig. 3:**
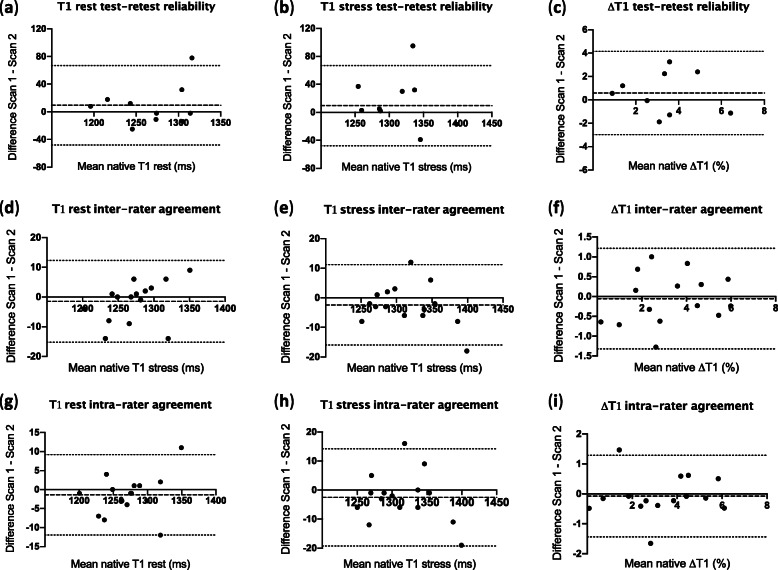
Bland-Altman plots for test-retest reliability of (**a**) resting native T1, (**b**) stress native T1, (**c**) ΔT1; interrater agreement of (**d**) resting native T1, (**e**) stress native T1, (**f**) ΔT1; intrarater agreement of (**g**) resting native T1, (**h**) stress native T1, (**i**) ΔT1

The mean interval between test and retest reliability scans was 7 ± 4 days. All ten patients had test-retest resting T1 maps as part of the reliability sub-study, but only nine underwent stress testing on the retest scan (one had caffeine). For test-retest scans, all T1 images were analysable. Image quality was either excellent (rest, *n* = 18; stress, *n* = 11) or moderate (rest, *n* = 2; stress, *n* = 4). The interrater and intrarater agreement of all parameters were excellent (ICC > 0.9). Test-retest reliability was good-excellent for rest and stress native T1, while it was moderate-poor for ΔT1 (Table 3 for global measurements and Table 4 for segmental measurements). Segmental values for ΔT1 test-retest reliability were not analysed given the already moderate-poor reliability of global measurements. Bland-Altman plots did not show evidence of systematic bias, although some data points were outside the 95% confidence interval (Fig. 3).

**Table 3:** **Test-retest reliability, interrater and intrarater agreement of global rest native T1, stress T1 and ΔT1**

Parameter	Study 1	Study 2	CoV	ICC (95% CI)	Bias ± SD of bias	BA Limits of Agreement
**Test-retest reliability (*n*** **= 10)**						
Rest T1	1273 ± 48	1264 ± 41	1.2%	0.79 (0.38; 0.94)	9.4 ± 29.3	−48.0; 66.8
Stress T1*	1322 ± 47	1305 ± 53	1.5%	0.69 (0.16; 0.92)	16.4 ± 38.3	−58.6; 91.5
ΔT1*	3.6 ± 1.8	3.0 ± 2.0	27.4%	0.55 (− 0.10; 0.88)	0.6 ± 1.8	−3.0; 4.1
**Interrater agreement (*n*** **= 15)**						
Rest T1	1272 ± 40	1274 ± 37	0.3%	0.98 (0.95; 0.99)	−1.5 ± 7.0	− 15.2; 12.3
Stress T1	1313 ± 44	1316 ± 46	0.3%	0.99 (0.96; 1.00)	− 2.4 ± 6.9	− 16.0; 11.2
ΔT1	3.2 ± 1.9	3.3 ± 1.7	9.6%	0.94 (0.83; 0.98)	−0.1 ± 0.6	−1.3; 1.2
**Intrarater agreement (*n*** **= 15)**						
Rest T1	1272 ± 40	1274 ± 38	0.2%	0.99 (0.97; 1.00)	−1.4 ± 5.4	−12.0; 9.2
Stress T1	1313 ± 44	1316 ± 46	0.3%	0.98 (0.95; 0.99)	− 2.5 ± 8.5	− 19.2; 14.2
ΔT1	3.2 ± 1.9	3.3 ± 1.8	10.3%	0.94 (0.83; 0.98)	−0.1 ± 0.7	−1.4; 1.3

Data presented as mean ± SD. * *n* = 9. *CoV* Coefficient of variability, *ICC* Intraclass correlation coefficient, *BA* Bland-Altman

**Table 4:** Test-retest reliability, interrater and intrarater agreement for individual mid-ventricular myocardial segments

Parameter	Study 1	Study 2	CoV	ICC (95% CI)	Bias ± SD of bias	BA Limits of Agreement
**Rest native T1 test-retest reliability (*n*** **= 10)**						
S1	1257 ± 56	1230 ± 56	2.6%	0.37 (−0.23–0.79)	26.1 ± 62.3	− 96.0; 148.2
S2	1232 ± 39	1226 ± 42	1.3%	0.69 (0.14–0.91)	5.4 ± 32.8	− 58.9; 69.7
S3*	1241 ± 38	1233 ± 33	1.2%	0.66 (0.08–0.91)	8.8 ± 29.4	48.8; 66.4
S4	1275 ± 57	1268 ± 48	2.0%	0.51 (− 0.17–0.85)	7.3 ± 53.2	− 96.9; 111.5
S5	1292 ± 50	1291 ± 44	0.9%	0.87 (0.54–0.97)	1.2 ± 26.7	− 49.1; 51.5
S6	1303 ± 54	1282 ± 47	2.4%	0.27 (− 0.37–0.74)	20.7 ± 61.2	− 99.2; 140.6
**Stress native T1 test-retest reliability (*n*** **= 9)**						
S1**	1300 ± 43	1269 ± 40	2.2%	0.24 (− 0.31–0.75)	31.5 ± 49.8	−66.1; 129.1
S2**	1266 ± 46	1229 ± 69	3.7%	− 0.19 (0.74–0.54)	37.0 ± 90.8	− 141.0; 215.0
S3	1298 ± 68	1262 ± 31	2.8%	0.19 (0.36–0.71)	35.7 ± 66.5	−94.8; 166.1
S4	1322 ± 56	1304 ± 86	3.0%	0.41 (− 0.33–0.83)	17.4 ± 80.4	−140.1; 175.0
S5	1326 ± 40	1345 ± 76	2.3%	0.50 (− 0.17–0.86)	−18.2 ± 61.1	− 138.0; 101.6
S6	1353 ± 53	1337 ± 41	1.8%	0.48 (− 0.18–0.85)	15.9 ± 47.9	−78.1; 109.8
**Rest native T1 interrater agreement (*n*** **= 15)**						
S1	1259 ± 47	1257 ± 46	0.4%	0.98 (0.94–0.99)	1.8 ± 9.4	− 16.6; 20.2
S2	1238 ± 33	1240 ± 33	0.7%	0.86 (0.67–0.96)	1.6 ± 17.0	− 34.8; 31.6
S3	1237 ± 37	1237 ± 30	0.4%	0.96 (0.89–0.99)	−0.6 ± 9.9	− 20.0; 18.8
S4	1269 ± 50	1273 ± 51	0.4%	0.98 (0.94–1.00)	− 4.5 ± 8.4	− 21.0; 12.1
S5	1294 ± 42	1296 ± 38	0.3%	0.98 (0.94–0.99)	−2.2 ± 8.3	− 18.4; 14.0
S6	1300 ± 44	1301 ± 42	0.4%	0.97 (0.91–0.99)	−1.1 ± 11.4	− 23.4; 21.2
**Stress native T1 interrater agreement (*n*** **= 15)**						
S1	1307 ± 52	1304 ± 66	1.0%	0.91 (0.76–0.97)	2.9 ± 25.7	− 47.5; 53.4
S2	1280 ± 57	1283 ± 57	0.4%	0.99 (0.96–1.00)	−2.8 ± 9.8	−22.0; 16.4
S3	1291 ± 59	1296 ± 64	0.5%	0.98 (0.93–0.99)	− 5.3 ± 12.2	−29.1; 18.6
S4	1312 ± 57	1316 ± 59	0.3%	0.99 (0.96–1.00)	−4.2 ± 8.4	−20.7; 12.3
S5	1329 ± 47	1326 ± 41	0.3%	0.98 (0.94–0.99)	3.2 ± 8.8	− 14.0; 20.4
S6	1339 ± 49	1344 ± 49	0.4%	0.97 (0.92–0.99)	−5.0 ± 10.3	− 25.2; 15.2
**ΔT1 interrater agreement (*n*** **= 15)**						
S1	3.8 ± 2.8	3.7 ± 3.1	29.8%	0.72 (0.34–0.90)	0.1 ± 2.3	−4.4; 4.6
S2	3.4 ± 3.7	3.5 ± 3.2	24.4%	0.88 (0.68–0.96)	− 0.1 ± 1.8	− 3.5; 3.4
S3	4.4 ± 3.7	4.7 ± 4.2	10.1%	0.97 (0.92–0.99)	− 0.4 ± 0.9	− 2.1; 1.4
S4	3.4 ± 2.1	3.4 ± 2.7	12.5%	0.94 (0.83–0.98)	0.0 ± 0.9	− 1.7; 1.7
S5	2.7 ± 2.0	2.3 ± 1.7	14.0%	0.93 (0.71–0.98)	0.4 ± 0.6	− 0.7; 1.6
S6	3.1 ± 2.0	3.4 ± 1.7	12.5%	0.91 (0.76–0.97)	−0.3 ± 0.8	− 1.8; 1.2
**Rest native T1 intrarater agreement (*n*** **= 15)**						
S1	1259 ± 47	1257 ± 49	0.3%	0.99 (0.97–1.00)	2.1 ± 7.0	− 11.6; 15.9
S2	1238 ± 33	1238 ± 36	0.3%	0.98 (0.95–0.99)	0.3 ± 6.4	− 12.3; 12.9
S3	1237 ± 37	1235 ± 34	0.3%	0.98 (0.95–0.99)	1.4 ± 6.5	−11.4; 14.2
S4	1269 ± 50	1274 ± 46	0.5%	0.97 (0.91–0.99)	− 4.7 ± 11.0	−26.3; 16.8
S5	1294 ± 42	1298 ± 41	0.4%	0.97 (0.92–0.99)	−4.1 ± 8.9	−21.5; 13.4
S6	1300 ± 44	1295 ± 40	0.4%	0.96 (0.90–0.99)	4.4 ± 10.8	− 16.8; 25.6
**Stress native T1 intrarater agreement (*n*** **= 15)**						
S1	1307 ± 52	1306 ± 68	0.9%	0.93 (0.79–0.98)	0.7 ± 24.2	−46.7; 48.1
S2	1280 ± 57	1280 ± 59	0.3%	0.99 (0.97–1.00)	0.5 ± 9.2	−17.6; 18.6
S3	1291 ± 59	1294 ± 61	0.3%	0.99 (0.98–1.00)	− 3.2 ± 6.5	− 15.9; 9.5
S4	1312 ± 57	1317 ± 58	0.5%	0.97 (0.92–0.99)	−5.5 ± 12.5	−30.0; 19.1
S5	1329 ± 47	1328 ± 39	0.5%	0.95 (0.86–0.98)	1.1 ± 14.0	− 26.3; 28.6
S6	1339 ± 49	1342 ± 50	0.4%	0.97 (0.92–0.99)	−2.4 ± 11.9	− 25.6; 20.8
**ΔT1 intrarater agreement (*n*** **= 15)**						
S1	3.8 ± 2.8	3.9 ± 2.8	22.5%	0.84 (0.59–0.94)	− 0.1 ± 1.8	− 3.6; 3.4
S2	3.4 ± 3.7	3.4 ± 3.4	12.3%	0.97 (0.92–0.99)	0.0 ± 0.9	− 1.7; 1.7
S3	4.4 ± 3.7	4.7 ± 3.7	9.6%	0.97 (0.92–0.99)	−0.4 ± 0.8	− 2.0; 1.3
S4	3.4 ± 2.1	3.4 ± 1.8	10.7%	0.93 (0.81–0.98)	0.0 ± 0.8	−1.5; 1.5
S5	2.7 ± 2.0	2.3 ± 1.8	20.1%	0.86 (0.63–0.95)	0.4 ± 1.0	−1.5; 2.3
S6	3.1 ± 2.0	3.6 ± 2.5	23.1%	0.76 (0.47–0.92)	− 0.5 ± 1.5	−3.5; 2.4

Data presented as mean ± SD. *n = 9; **n = 8. *CoV* Coefficient of variability, *ICC* Intraclass correlation coefficient, *BA* Bland-Altman

### Associations between clinical variables and myocardial stress T1 reactivity

**Fig. 4:**
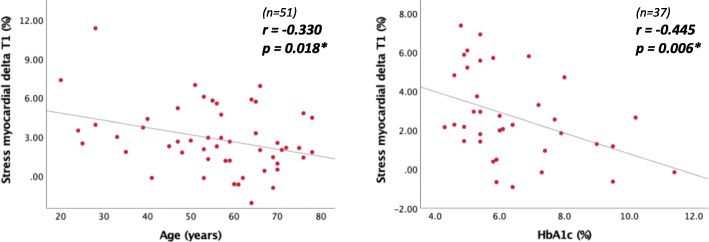
Scatter plots displaying correlations between (left) ΔT1 and age; (right) ΔT1 and glycosylated haemoglobin (HbA1c)

**Fig. 5:**
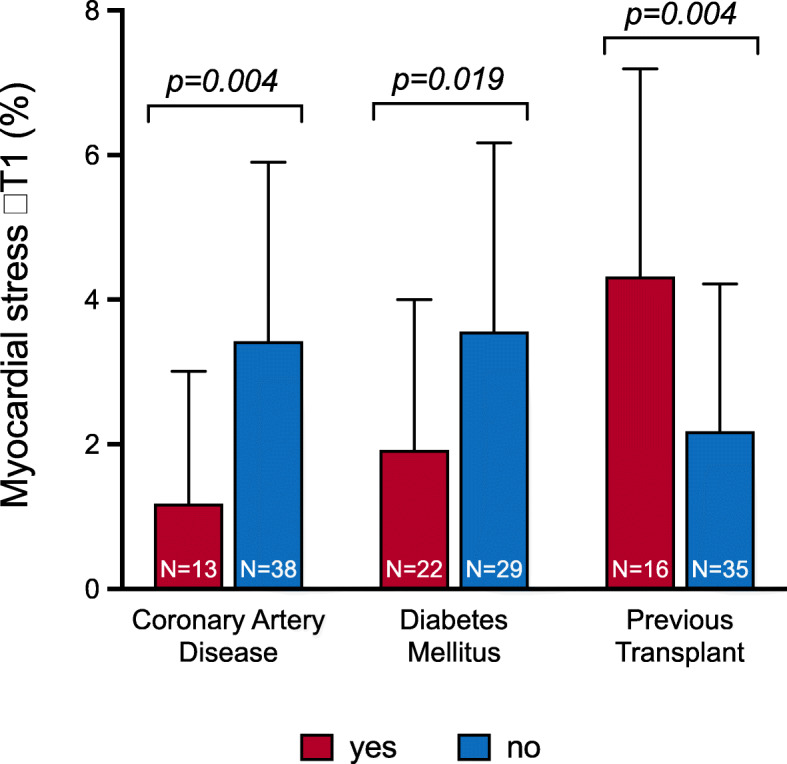
Differences in myocardial stress T1 reactivity (ΔT1) between groups. ΔT1 was significantly lower in patients with coronary artery disease; diabetes mellitus compared to patients without these pathologies. Patient who previously had a kidney transplant displayed significantly higher ΔT1 compared to patients who had only been on dialysis

For the 51 participants with analysable stress T1 maps, an exploratory analysis was conducted to establish associations between ΔT1 and clinical characteristics. ΔT1 was not significantly different between males and females (*p* = 0.51). ΔT1 decreased with increasing age and increasing haemoglobin A1c (HbA1c) (Fig. 4). ΔT1 was blunted in patients with evidence of CAD (1.2% ± 1.8 versus 3.4% ± 2.5, *p* = 0.004) or diabetes (1.9% ± 2.1 versus 3.6% ± 2.6; *p* = 0.019) (Fig. 5). Patients who had a previous kidney transplant showed a higher ΔT1 compared to those who solely had dialysis as RRT (4.3% ± 2.9 versus 2.2% ± 2.0; *p* = 0.004) (Fig. 5). However, the proportion of total RRT time spent with a functioning kidney transplant as opposed to dialysis was not significantly associated with ΔT1. There was no clear relationship between ΔT1 in all-time smokers compared to non-smokers (2.4% ± 2.4 versus 3.3% ± 2.8; *p* = 0.23). ΔT1 was not significantly different in patients with or without hypertension or dyslipidaemia. ΔT1 was not significantly associated with HR and BP. ΔT1 was inversely related to resting native T1 (r = − 0.533; *p* < 0.01) but it was not significantly correlated with stress native T1.

On multivariate analysis, CAD, diabetes and resting native T1 remained independent determinants of ΔT1, after adjustment for age and proportion of total RRT time with a functioning graft (Table 5).

**Table 5:** **Multivariate linear regression model for the independent predictors of myocardial T1 reactivity (ΔT1)**

	B (95% CI)	Beta	*P* value
Resting native T1 (ms)	−0.020 (− 0.031; − 0.010)	−0.455	< 0.001*
Diabetes mellitus	−1.488 (− 2.647; − 0.330)	− 0.297	0.013*
Coronary artery disease	−1.570 (− 2.865; − 0.276)	−0.275	0.019*
Age (years)	−0.010 (− 0.050; 0.030)	−0.058	0.627
% total RRT time with transplant	0.006 (−0.013; 0.025)	0.071	0.540

Linear regression model R^2^ = 0.484, adjusted R^2^ = 0.427. B = unstandardised regression coefficient. Beta = standardised regression coefficient. *statistically significant, *p* < 0.05. *Abbreviations*: *RRT* Renal replacement therapy

### Regional wall motion scores and ΔT1

For the 51 participants with analysable stress T1 maps 306 segments were analysed on corresponding mid-ventricular LV cine images. 279 segments were scored ‘1’, 19 segments were scored ‘2’ and 8 segments were scored ‘3’. All but one of the segments scored ‘3’ corresponded to an area on T1 maps identified as infarct which was too thin to contour. Mean ΔT1 in segments scored ‘1’ was 3.01% (3.5), mean ΔT1 in segments scored ‘2’ was 1.6% (3.4) and the only segment scored ‘3’ with an analysable T1 maps had a ΔT1 of 1.5%.

## Discussion

This is the first study to assess the feasibility of stress T1 mapping in patients on haemodialysis. We have shown that adenosine stress T1 mapping is a safe, feasible and well-tolerated technique for the potential assessment of myocardial ischaemia in a cohort of 58 patients with ESRD on haemodialysis and that there were no adverse events associated with its use. We have shown native T1 mapping at rest and during stress to be a highly reproducible technique and have described the reliability and agreement of ΔT1 for the first time in this population, which was moderate. ΔT1 was independently associated with CAD, diabetes and resting native T1.

Our group has previously demonstrated the excellent reliability of rest native T1 mapping in patients with ESRD, independent of fluid status [21]. In our study, ΔT1 appears to be a less reliable measure. This may be attributable to the methodology for ΔT1 calculation, as it is based on two independent measurements, amplifying the possibility of variability. Liu et al. assessed reliability with Bland-Altman analysis [12], therefore our study is the first to describe ΔT1 reliability in terms of CoV and ICC, precluding direct comparison of these values with previously reported data. The test-retest bias on Bland-Altman analysis for ΔT1 we report (0.6% ± 1.8) is poorer compared to that described by Liu et al. (0.18% ± 0.36) [12]. This discrepancy might be attributable to differences in the selection of patients, as our study focusses on prevalent ESRD patients, some with comorbidities such as CAD, hypertension and diabetes, whereas Liu et al. assessed ΔT1 reliability in healthy subjects [12]. The difference in acquisition technique (MOLLI versus ShMOLLI) might also contribute to the increased variability, however a head-to-head comparison of the two techniques is needed to confirm this. Cardiac and respiratory motion remain a concern for MOLLI-based acquisition during stress as they lead to loss of spatial resolution, in the presence of high HR, RR variability and residual respiratory movement, which are not always amenable to inline motion correction [24, 27]. The lack of a significant correlation between HR and native T1 values at rest or during stress in our study suggests HR did not impact T1 value itself, despite the HR sensitivity of the classic MOLLI 3(3)3(3)5 technique [19]. This is probably due to the fact that only rarely the HR during stress was above 100 bpm, where the MOLLI sequence is known to be less reliable. When compared to the test-retest reliability reported for the assessment of myocardial perfusion reserve on CMR by semi-quantitative (myocardial perfusion reserve index) or quantitative (myocardial perfusion reserve) analysis, ΔT1 as a method for assessment of myocardial blood volume change performs comparably. Test-retest reliability of myocardial perfusion reserve index has been reported to have a CoV of 19 to 27% in healthy subjects and patients with CAD [28, 29]. For myocardial perfusion reserve, studies have reported test-retest CoVs varying from of 13.3 to 35% [29, 30] in healthy subjects and ICCs varying from 0.26 to 0.88 [30, 31].

We have shown the mean ΔT1 for this population is 2.9 ± 2.5%, substantially below the ΔT1 previously reported in healthy population (6.3 ± 1.1%) [10] and equivalent to that for myocardium with microvascular dysfunction (3.0 ± 0.9%) [12]. This is as expected as patients with ESRD on haemodialysis are known to have a high preponderance of micro and macrovascular dysfunction. Moreover, ΔT1 appears to be able to differentiate between patients with and without CAD and diabetes, further demonstrating the potential clinical application of the technique. Our finding that a history of CAD and diabetes were both independent determinants of ΔT1 in multivariate analysis is in keeping with the blunted ΔT1 reported in the presence of macrovascular epicardial stenosis [10, 12] or microvascular disease [12, 23]. However, although our study does suggest that the presence of CAD or diabetes are clinical determinants a blunted ΔT1, the wide standard deviations we report in this study question the applicability of the ΔT1 thresholds proposed by Liu et al. to detect obstructive CAD and microvascular dysfunction (1.5 and 4.0%, respectively) [12] to patients with ESRD. The description of putative differences between segmental RWM scores and ΔT1 should be viewed as exploratory and hypothesis generating but do not contradict the hypothesis that ΔT1 is blunted in areas of reduced myocardial blood supply.

We acknowledge that we have analysed ΔT1 of the whole mid-ventricular short axis slice, as opposed to individual coronary territories meaning that the ΔT1 might average across normal and non-normal segments, possibly leading to a higher degree of variation. We also found that increased resting myocardial native T1 was an independent determinant of a blunted ΔT1 amongst patients with ESRD. Two possible explanations may account for this finding. Firstly, a higher resting native T1 might signify increased levels of myocardial fibrosis or other pathological processes related to excess water, such as oedema or inflammation [6]. Perivascular myocardial fibrosis is known to predispose to diminished coronary reserve [32, 33] and it could, therefore, limit the increase in myocardial blood volume and result in a blunted ΔT1. Secondly, an elevated resting native T1 could indicate compensatory microcirculatory vasodilation downstream of obstructive epicardial coronary stenosis to preserve myocardial oxygen supply [34]. This would cause an expansion of the intravascular space and subsequent myocardial blood volume increase [35, 36], reflected in the elevated resting T1, in keeping with what was observed by Liu et al. [10, 12]. Our study did find a trend towards higher resting native T1 amongst patients with CAD compared to patients without (1279 ms ± 40 versus 1255 ms ± 60), however, this was not statistically significant, possibly due to inadequate sample size and a type II error. The lack of a trend towards higher resting native T1 in individuals with DM possibly suggests that microvascular dysfunction does not cause a similar expansion of baseline intravascular volume and a subsequent elevation of resting native T1, in keeping with the results of previous studies [10, 12, 37].

With regards to concerns of the influence of body water content on native T1 measurement, our group have previously demonstrated that myocardial native T1 was unaffected by changes in fluid status [21]. Furthermore, a subsequent study assessed changes in myocardial native T1 pre and post haemodialysis. Whilst they found a statistically significant difference, the absolute mean change in native T1 was only 13 ms, representing approximately a 1% change [38].

### Study limitations

The cross-sectional design of this feasibility study precludes from any causal relationship to be inferred from the findings we present and does not allow the assessment of clinical endpoints including cardiac events and mortality. Our study was conducted at 3 T using the classic MOLLI sequence, therefore our findings in terms of reliability are not necessarily generalisable to a 1.5 T platform, other MOLLI variants or saturation-recovery single-shot acquisition sequences. Despite not finding a significant correlation between HR and T1 values, we recognise that the HR dependency of the MOLLI sequence may have led to underestimation of stress T1 values and, consequently ΔT1, although maximum HR was rarely elevated above 100 bpm. While this study offers important information with regards to the feasibility, reliability and clinical determinants of ΔT1 in patients with ESRD, the lack of basal and apical slices meant that we could not build the complete American Heart Association 16-segment model for analysis of individual coronary territories and global myocardium. We were unable to provide a direct comparison of stress T1 mapping findings against invasive coronary angiography or non-invasive investigations for CAD, as there was no simultaneous screening for CAD, meaning that we cannot be sure we captured all patients with CAD. This also meant that CAD was assessed as a binary variable, without taking into account extent or severity of disease. Additionally, although obviously infarcted segments of myocardium were excluded from analysis, we acknowledge small areas of replacement fibrosis might not have been detected and, therefore, excluded. As high levels of replacement fibrosis would account for a blunted ΔT1, this is another reason why direct comparison with established investigations for CAD is required.

## Conclusions

Rest-vasodilator stress native T1 mapping is feasible in ESRD although test-retest reliability is moderate. These data support the development of an adequately powered prospective study to evaluate the diagnostic accuracy of stress T1 mapping in an ESRD population through direct comparison to invasive coronary angiography and non-invasive measures of CAD.

## Data Availability

The datasets generated and/or analysed during the current study are not publicly available but are available from the corresponding author on reasonable request.

## Appendix 1

**Table:** Table 6

	*n* = 58
Ever smoked, n (%)	29 (53)
Pack years	1 (0; 25)
Medication data	
Erythropoietin (units/week)	6000 (1000; 9000)
Intravenous iron (mg/week)	0.0 (0.0–50.0)
ACE inhibitors, n (%)	5 (8.6)
ARB, n (%)	7 (12.1)
Beta-blockers, n (%)	26 (44.8)
Statins, n (%)	35 (60.3)
Calcium-channel blockers, n (%)	27 (46.6)
Diuretics, n (%)	17 (29.3)
Alpha blockers, n (%)	11 (19.0)
Aspirin, n (%)	24 (41.4)
Insulin, n (%)	14 (24.1)
Oral hypoglycaemic, n (%)	6 (1.0)
Haematological and biochemical data	
Sodium (mmol/L)	136.6 ± 3.2
Potassium (mmol/L)	5.0 ± 0.9
Bicarbonate (mmol(L)	25.3 ± 3.0
Phosphate (mmol/L)	1.5 (1.3; 2.0)
Calcium (mmol/L)	2.3 ± 0.2
Urea reduction ratio (%)	76.5 (70.8; 81.3)
Haemoglobin (g/L)	111.5 (99.0; 123.0)
Total cholesterol (mmol/L) *[n = 45]*	3.8 (3.0; 4.5)
HbA1c (%) *[n = 43]*	5.8 (5.1; 7.2)
WCC (10^9^/L)	6.3 (5.3; 8.8)
Platelets (10^9^/L)	215.3 ± 82.8
Albumin (g/L)	36.8 ± 5.2
Ferritin (ng/ml) *[n = 57]*	278 (176; 403)
CRP detectable (≥5) *[n = 45]*	22
CRP (mg/L) *[n = 22]*	18 (12; 36)
PTH [*n* = 37]	49 (17; 82)

Categorical variables presented as number (%). Normally distributed data presented as mean ± SD. Non-normally distributed data presented as median (P25, 75)

*ACE* Angiotensin-converting-enzyme, *ARB* Angiotensin-receptor blocker, *BMI* Body mass index, *CRP* C-reactive protein, *HbA1c* Glycosylated haemoglobin, *PTH* Parathyroid hormone, *WCC* White cell count

## Abbreviations

BP: Blood pressure
CAD: Coronary artery disease
CMR: Cardiovascular magnetic resonance
CoV: Coefficient of variability
ECG: Electrocardiogram
ESRD: End stage renal disease
HR: Heart rate
ICC: Intraclass correlation coefficient
LV: Left ventricle/left ventricular
MOLLI: Modified Look-Locker inversion recovery
RRT: Renal replacement therapy
RWM: Regional wall motion
ΔT1: Myocardial stress T1 reactivity
